# A Rapidly Debilitating Myopathy: A Rare Case of Statin-Induced Necrotizing Myositis

**DOI:** 10.7759/cureus.16304

**Published:** 2021-07-10

**Authors:** Anam Ahmad, Imad Karam, Donica L Baker

**Affiliations:** 1 Internal Medicine, St. Luke's Hospital, Chesterfield, USA; 2 Rheumatology, St. Luke's Hospital, Chesterfield, USA

**Keywords:** statin-induced necrotizing autoimmune myositis, autoimmune necrotizing myositis, statins, statin-induced myositis, statin-induced rhabdomyolosis

## Abstract

Statins are well tolerated in general but can be associated with myopathies. Statin-induced myopathies can range widely from mild myalgias to necrotizing autoimmune myopathies. We present a case of an 81-year-old man on statins for five years with no complications, who developed progressive muscle weakness, rhabdomyolysis, and dysphagia. His laboratory workup revealed elevated inflammatory markers with creatine kinase (CK) levels above 2000 U/L. The myositis panel was negative, and the anti-3-hydroxy-3-methylglutaryl-coenzyme A reductase antibody was positive. His muscle biopsy showed randomly scattered necrotic fibers with minimal perivascular inflammation confirming statin-induced necrotizing autoimmune myopathy (SINAM). Statins were discontinued immediately after initial suspicion. The patient was started on intravenous immunoglobulin followed by hydrocortisone and mycophenolate mofetil. The patient continued to have muscle weakness and progressive dysphagia to the point that he could not handle his secretions. His disease course was complicated by recurrent aspiration pneumonia. Percutaneous endoscopic gastrostomy tube placement was considered, but his family decided on hospice care given his overall comorbidities. Physicians should note that SINAM can occur after a few months to several years of statin use. This disease can be rapidly debilitating and progress even after discontinuation of statins, and treatment requires immunosuppressants, including steroids and steroid-sparing agents.

## Introduction

Statins are commonly used to reduce cardiovascular risks. Generally, they are well tolerated, but muscle-related adverse effects are common and range from mild myalgias to necrotizing autoimmune myopathies [1,2]. Necrotizing autoimmune myositis is rare and possibly associated with connective tissue disorders, cancer, and drugs, such as statins [3]. It is characterized by acute or subacute muscle weakness along with myocyte necrosis without inflammation on histopathology. It can occur from a few months to even years after the use of statins. Diagnosis is based on biopsy, and treatment includes high-dose glucocorticoids, steroid-sparing agents, and intravenous immunoglobulins (IVIG). We present a case of statin-induced rhabdomyolysis and necrotizing autoimmune myositis occurring five years after using statins.

## Case presentation

An 81-year-old man was admitted from a skilled nursing facility (SNF) due to severe generalized fatigue, elevated creatine kinase (CK) levels above 2000 U/L, and elevated creatinine of 1.6 mg/dL. His medical history was significant for coronary artery disease with cardiac stent placement two months before admission. His history was also significant for diabetes, hypertension, herniated lumbar disc-related back pain, and dysphagia.

The patient was in a normal state of health two months before admission and used to play golf three times per week. According to his family, following his cardiac stent placement two months ago at an outside facility, his condition worsened. He developed proximal muscle weakness, generalized deconditioning, dysphagia, and he sustained multiple falls. The patient was admitted to our facility one week ago with shortness of breath. He was admitted and subsequently treated with antibiotics due to suspicion of aspiration pneumonia. During that hospitalization, we conducted an esophagogastroduodenoscopy as a workup of dysphagia with dilatation of the esophagus. His liver enzymes were also found to be elevated, and his gallbladder was distended in the absence of a gallstone. His liver function test results were thought to be elevated due to rosuvastatin, and the medication was stopped. The patient was discharged to rehabilitation after treatment of aspiration pneumonia. He was also on a dexamethasone tapering dose for his chronic back pain and shortness of breath.

At the SNF, the patient could not participate in therapy due to muscle weakness and could not eat solid foods because of progressive dysphagia. He was readmitted for generalized fatigue and elevated CK and creatinine levels, as noted. Diffuse muscle weakness strength (Grades 3 to 4/5 in his lower extremities) was noted. His straight leg raising test was positive bilaterally. His laboratory workup was significant for elevated erythrocyte sedimentation rate (28 mm/h), aldolase levels (19 U/L), and CK levels (2315 U/L). The initial differential diagnosis included rhabdomyolysis, dermatomyositis, polymyositis, steroid-induced myopathy, and statin-induced myopathy. He was started on intravenous (IV) hydration.

**Figure 1:**
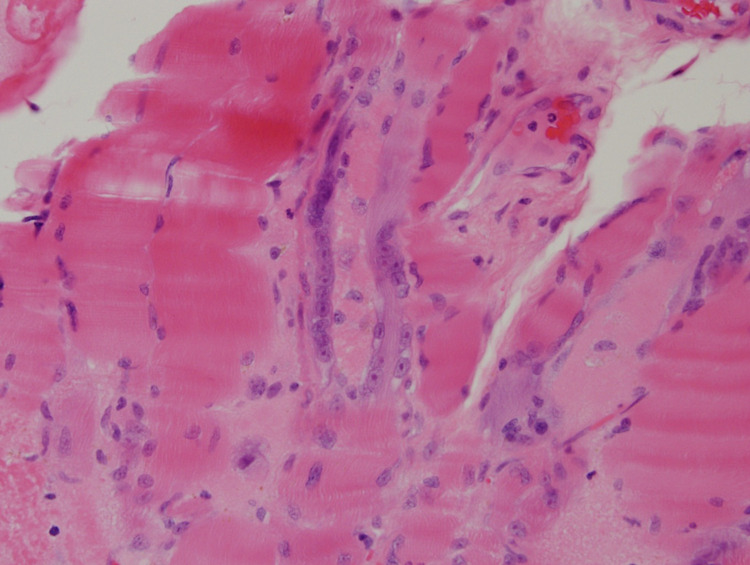
Cross-section of skeletal muscle with a cluster of pale eosinophilic necrotic fibers surrounded by normal-sized and atrophic fibers. A regenerating fiber lies at the tissue edge to the left of the center. Note the absence of inflammation.

**Figure 2:**
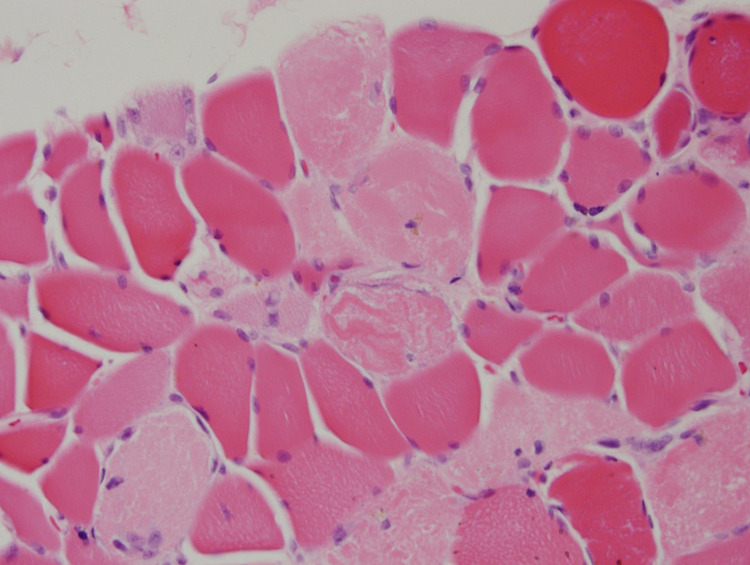
Tangentially cut myofiber in the center with a sausage-shaped area of central necrosis surrounded by a rim or regenerative activity. Adjacent normal, necrotic, and regenerating fibers are seen.

Considering his muscle weakness and elevated CK levels, a rheumatologist was consulted. The patient underwent a workup, including myositis panel, extractable nuclear antigen antibodies panel, anti-double-stranded DNA antibody test, anti-histidyl-transfer ribonucleic acid synthetase antibody test, and anti-3-hydroxy-3-methylglutaryl-coenzyme A (anti-HMG-CoA) reductase antibody test. All test results were negative except his anti-HMG-CoA reductase antibody levels (28.7 U/mL), raising high suspicion of necrotizing autoimmune myositis. The patient’s statin was halted one week prior during a recent hospitalization. A muscle biopsy showed many randomly scattered necrotic fibers and some underlying phagocytosis but no vasculitis or focal endometrial fibrosis (Figures 1, 2). No endometrial or chronic perivascular inflammation was found, which confirmed the diagnosis of statin-induced necrotizing autoimmune myositis (SINAM). The patient was initially started on IVIG (25 mg daily) based on body weight for three days, followed by hydrocortisone (100 mg every eight hours) and mycophenolate mofetil (500 mg twice daily). He continued to have progressive dysphagia that worsened to the point that he had difficulty managing his secretions. The hospital course was complicated by recurrent aspiration pneumonia and thrombocytopenia. Due to his condition and multiple comorbidities, the family decided on hospice care. The patient was taken off mycophenolate mofetil and discharged to hospice care.

## Discussion

Statins act on HMG-CoA reductase, a rate-limiting enzyme in the cholesterol biosynthesis pathway. SINAM has a prevalence of one in 100,000 [4] and is characterized by proximal muscle weakness with marked serum CK elevations and histological evidence of myonecrosis, with little or no inflammatory cell infiltration. SINAM is associated with the presence of autoantibodies directed against HMG-CoA reductase [4].

Generally, SINAM is more prevalent in patients who are positive for the human leukocyte antigen-DR isotype [3,4]. The type of statin used is also a factor, as lovastatin, simvastatin, and atorvastatin have more effects on muscles. Dose strength is another risk factor, as the higher dose of the statins is associated with a greater risk of muscle injury. As hepatic cytochrome P450 3A4 (CYP3A4) metabolizes statins, patients on other drugs that affect the metabolism of CYP3A4 like macrolides, azoles, cyclosporine, colchicine, calcium channel blockers, fibrates, and amiodarone are more prone to SINAM [4,5]. Patients who have other underlying neuromuscular and muscular dysfunctions are also more prone to SINAM. The onset of the condition is insidious and can vary. Our patient tolerated rosuvastatin well for the last five years, then suddenly and rapidly experience progressive muscle weakening.

Pathogenesis involves overexpression of the gene encoding HMG-CoA reductase in genetically predisposed people either in the presence or absence of other environmental risk factors [4]. It is still unknown whether these antibodies directly affect the myocytes or accelerate complementary pathways. Still, once the pathway has been triggered, it propagates a positive biofeedback loop [5].

Immune-mediated necrotizing myopathy generally presents as progressive symmetrical proximal muscle weakness and myalgias. In some cases, the patient experiences respiratory and esophageal muscle weakness (as seen in our patient) and dysphagia [6]. Diagnosis requires an adequate history of statin exposure, physical examination, and laboratory testing. On physical examination, we might note reduced muscle strength in proximal muscles. The initial evaluation should exclude other causes of muscle weakness like Cushing’s disease, Addison’s disease, hypothyroidism, other neuromuscular diseases, and muscular dystrophies.

For laboratory tests, CK levels, renal function tests, and urine analysis for myoglobinuria should be considered [7]. Creatine phosphokinase (CPK) levels are usually very high (>1000 IU/L) in SINAM cases (as seen in our patient). For a definite diagnosis, positive anti-HMG-CoA reductase antibodies and muscle biopsies are required. However, anti-HMG-CoA reductase can be present without prior statin exposure in some patients. Still, statin exposure and high CK levels correlate with the clinical severity of the statin-induced autoimmune disease [8]. MRI can demonstrate muscle atrophy and intramuscular edema. Muscle biopsy predominately shows myofiber necrosis with relatively mild inflammation and a lack of lymphocyte infiltration [8]. Instead, macrophages are present in abundance, engulfing necrotic muscle fibers, noted in our case. 

Management includes immediate discontinuation of statins and immunosuppressants. Oral high-dose steroids and IVIG are first-line therapies [9]. Other adjuvant therapies like methotrexate, azathioprine, and rituximab can also be considered. Monitoring CK levels and falling antibody levels correlate with improved outcomes [10]. Tapering the dose of immunosuppression can be challenging in some cases because SINAM can relapse [11]. Other newer agents like ezetimibe and proprotein convertase subtilisin/kexin type 9 inhibitors can be used for long-term management of hypercholesteremia in these patients. The disease can still progress even after stopping statins, as noted in our patient.

## Conclusions

Physicians should consider the onset of SINAM when patients treated with statins present with myalgias, proximal muscle weakness, and very high CPK levels even after chronic use of statins. Also, physicians should screen for antibodies against HMG-CoA reductase and stop statin therapy immediately. Patients should be started on steroids as the disease can progress even after discontinuation of the statins and can be debilitating if it involves respiratory muscles and esophageal muscles. As it has a genetic predisposition, future studies are required to develop a strategy to stratify people at a high risk of developing SINAM to avoid significant morbidity.
